# Effect and cost of two successive home visits to increase HIV testing coverage: a prospective study in Lesotho, Southern Africa

**DOI:** 10.1186/s12889-019-7784-z

**Published:** 2019-11-01

**Authors:** Niklaus Daniel Labhardt, Isaac Ringera, Thabo Ishmael Lejone, Alain Amstutz, Thomas Klimkait, Josephine Muhairwe, Tracy Renee Glass

**Affiliations:** 1 Department of Medicine, Swiss Tropical and Public Health Institute, Basel, Switzerland; 2 Infectious Diseases and Hospital Epidemiology, University Hospital Basel, Basel, Switzerland; 3 University of Basel, Basel, Switzerland; 4 SolidarMed, Swiss Organization for Health in Africa, Maseru, Lesotho; 5 Molecular Virology, Department of Biomedicine, Basel, Switzerland

**Keywords:** HIV, Home-based, Testing, Door-to-door, Coverage, Community-based testing

## Abstract

**Background:**

Home-based HIV testing and counselling (HB-HTC) is frequently used to increase awareness of HIV status in sub-Saharan Africa. Whereas acceptance of HB-HTC is usually high, testing coverage may remain low due to household members being absent during the home visits. This study assessed whether two consecutive visits, one during the week, one on the weekend, increase coverage.

**Methods:**

The study was a predefined nested-study of the CASCADE-trial protocol and conducted in 62 randomly selected villages and 17 urban areas in Butha-Buthe district, Lesotho. HB-HTC teams visited each village/urban area twice: first during a weekday, followed by a weekend visit to catch-up for household members absent during the week. Primary outcome was HTC coverage after first and second visit. Coverage was defined as all individuals who knew their HIV status out of all household members (present and absent).

**Results:**

HB-HTC teams visited 6665 households with 18,286 household members. At first visit, 69.2 and 75.4% of household members were encountered in rural and urban households respectively (*p* < 0.001) and acceptance for testing was 88.5% in rural and 79.5% in urban areas (p < 0.001), resulting in a coverage of 61.8 and 61.5%, respectively. After catch-up visit, the HTC coverage increased to 71.9% in rural and 69.4% in urban areas. The number of first time testers was higher at the second visit (47% versus 35%, p < 0.001). Direct cost per person tested and per person tested HIV positive were lower during weekdays (10.50 and 335 USD) than during weekends (20 and 1056 USD).

**Conclusions:**

A catch-up visit on weekends increased the proportion of persons knowing their HIV status from 62 to 71% and reached more first-time testers. However, cost per person tested during catch-up visits was nearly twice the cost during first visit.

**Trial registration:**

NCT02692027 (prospectively registered on February 21, 2016).

## Background

High coverage of antiretroviral therapy (ART) to achieve viral suppression in people living with HIV/AIDS is a cornerstone of the UNAIDS strategy to end AIDS by 2030 and is defined in the 90–90-90 targets [1]. Universal awareness of HIV status through broad HIV testing is the front door to this strategy [2]. In South-Eastern Africa, the region most heavily affected by the HIV epidemic, the percentage of people living with HIV who know their status has been steadily increasing over the last years and, according to UNAIDS, by the end of 2017 was estimated 81% [3].

Providing home-based HIV testing and counselling (HTC) is a well-established approach to reach individuals who do not seek testing at health facilities and to increase testing coverage in the population [4, 5]. In a meta-analysis that included 31 studies of home-based HTC, pooled uptake – acceptance of HTC – was 82% [6]. However, even where uptake may be high, coverage often remains low as home-based HTC misses all those individuals who are absent during the home visit. This may particularly affect working household members and children and adolescents going to school since most home-based HTC campaigns are conducted during regular working-hours. In their meta-analysis, Sharma and colleagues estimated coverage achieved through home-based HTC. Of the identified 16 studies reporting on HTC coverage and conducted after 2005, nine were of large scale (> 10′000 individuals) [6]. Coverage rates varied widely from 33% in a study conducted in Malawi [7] to 96% in study in Kenya [8]. More recently, the SEARCH project, which used a hybrid approach of mobile clinics and home-based testing, achieved 91 and 87% testing coverage in Ugandan and Kenyan regions, respectively [9].

Lesotho, a small landlocked country surrounded by South Africa, has the second-highest HIV prevalence globally (adult HIV prevalence 23.6 [21.2–24.7]) [10]. In 2014, 63% of adult women and 38% of men reported having had an HIV test in the last 12 months, and according to 2017 UNAIDS estimates 72% were aware of their status in 2016 [11]. In a cluster-randomized trial, home-based HTC had an acceptance rate of 92.5%. However, this trial did not assess coverage, and the rather low HIV prevalence among those tested through home-based HTC suggests that mostly household members with lower risk of exposure had been reached in this trial [12].

Here, we report HTC coverage achieved through a new home-based HTC strategy where households are visited twice – first on a workday and then on a weekend day in order to catch household members absent during the week.

## Methods

### Design

We report the results of a sub-study of the CASCADE-trial [13]. The here reported sub-study is a predefined nested study of the published protocol of the CASCADE-trial [14]. The major objective of this cross-sectional study is to assess coverage achieved through a two-day home-based HTC. The first visit always occurred during a work day. The second visit was strictly on weekends and aimed at offering HTC to household members who were absent during the first visit. The home-based HTC campaigns were conducted in rural and semi-urban neighborhoods of six catchment areas in Butha-Buthe district in northern Lesotho. For rural areas, villages were randomly selected from a list of eligible villages. To be eligible, a village had to be clearly confined to one of the six catchment areas and to comprise between 40 and 80 households. Catchment areas of the clinics are defined by the district health authorities. In Butha-Buthe town, urban neighborhoods were randomly selected.

### Setting

Butha-Buthe is a mostly rural district with an estimated 110,000 habitants, primarily subsistence farmers, mine workers, or construction and domestic workers in neighboring South Africa. The district has only one small town (Buthe-Buthe, ca. 25,000 habitants), while the remaining habitants live in villages scattered over a partly mountainous surface of 1767 km^2^. In 2016, the adult HIV-prevalence (15–49 years) was 17.8% [15]. The district is served by one public, one missionary hospital and 10 health centers. Similar to other high prevalence settings, HTC is provided by lay counsellors [16]. Lay counsellors are persons who completed at least primary education and underwent a standardized 10-day training on how to conduct HTC. They are supervised by a professional counsellor with tertiary education. In our study all participating lay-counsellors were female.

### Procedure

Chiefs of the selected villages and urban neighborhoods were informed prior to the home-visits. From February 22 to July 17, 2016 three teams, each consisting of four lay-counsellors, one professional counsellor, one nurse and a driver, visited one village per day. Upon arrival the team members divided up the village assigning a specific area to each lay-counsellor. Lay-counsellors then walked from house to house to propose HTC to all households in their area. The head of household or a substitute (usually the oldest household member present) had to provide written consent before the lay-counsellor could enter. Once inside and behind closed doors, the lay-counsellor first counted all members living in the respective household – present and absent. Every person spending at least two nights per month in the household was defined as household member. This definition was chosen because in Lesotho many family members work in neighboring South Africa and return back home only 1 week-end per month. Once information about all members was collected, the lay-counsellor proposed HTC to all individuals who were present. HTC was performed according to international and national guidelines [17]. All household members who were not known to be HIV-positive were eligible for HTC. As per guidelines, each individual had to sign the National HTC consent form before being tested. For children below 12 years of age, a caregiver aged ≥21 years had to provide consent. Household members could choose to test in family or separately. Initial testing was done with Alere Determine™ HIV-1/2. In case of a positive result, Trinity Biotech Uni-Gold™ was used for confirmatory testing. Once all who accepted HTC were tested, the lay-counsellor announced the week-end day he/she would return to propose HTC to absent household-members. This second visit was always during the following weekend, based on a list with households where members had been absent at first visit. Each lay-counsellor was re-assigned to the same households she had seen during the first round. Members absent at first visit but present at the second visit were proposed HTC. Persons found HIV positive were assessed for eligibility for the CASCADE-trial as outlined in the protocol [14]. Those not eligible were assessed by the nurse and referred to the clinic.

### Data and analysis

The major variable of interest was the HTC coverage achieved in the study areas after first and second visit of home-based HTC. Coverage was defined as the percentage of individuals knowing their HIV status after the home visits (either already known to be HIV infected or tested at one of the two home-visits during the study) out of all household members (encountered and not encountered during the two home-visits). HTC uptake was defined as follows: number of individuals who accepted HTC over all household members encountered at home and who were not known to be HIV positive. In addition to coverage and uptake we assessed time since last test categorizing clients into “never tested before”, “tested ≥12 months ago” and “tested <12 months ago”.

Data-collection was tablet-based using a specifically developed app (Visible Solutions AG, Switzerland, visibleimpact.org). For every household member, the outcome of the HTC home visit was categorized into “not encountered”, “encountered, already known to be HIV-positive”, “encountered, refused HTC” and “encountered, took up HTC”.

Every evening, when the study team had returned to town, data were uploaded from tablets to a banking-standard ISO 27001 audited data center and deleted from the mobile device.

### Statistical analysis

Data were summarized with frequencies and percentages for categorical data and medians and interquartile ranges (IQR) for continuous data. Categorical data were compared with chi-square tests and continuous data with Wilcoxon rank sum tests.

Subgroups of apriori interest were children (age < 15 years of age), male adults, and female adults. Univariable logistic regression models were utilized to test for the association between these three subgroups and presence at home, uptake and coverage. Within the three subgroups, the association between previous testing status and uptake and HIV prevalence was modeled using logistic regression. Results are presented with odds ratios (OR) and 95% confidence intervals (CI). For HTC uptake and coverage, we further stratified analysis by urban versus rural areas. We further stratified outcome analyses All data analysis was done using Stata version 14 (StataCorp, College Station, TX, USA).

### Cost considerations

In this article we report the retrospective cost-analysis considering direct cost of staff, staff training, equipment and transport while planning and conducting the HTC campaigns. Transport cost included fuel, vehicle insurance, tire replacement, car servicing and repair but not the purchase of the vehicle. The sum of all costs was then divided by the number of persons tested or number of persons newly tested positive. Cost attributed per item is reported in the Additional file 1: Table S1.

## Results

### Participants

From February 22 to July 17, 2016 counsellors visited 62 rural villages and 17 urban areas. Out of a total of 6660 occupied households, 5 (0.08%) heads of household did not consent to a household visit. The 3800 consenting rural households (median 50 (IQR 27–80) per village) had 11,368 household members (median 3, IQR 1–4 per household). In the urban areas, the 2855 consenting households (median 121 (IQR 67–273) per urban area) had 6968 household members (median 2, IQR 1–4 per household).

### Coverage after first visit

During the first household visit, 69.2% of the household members in villages and 75.4% of those in urban areas were at home (*p* < 0.001). Among all 13,119 household members encountered at home during first visit, 1365 (10.4%) were already known to be HIV-infected. Among the remaining 11,754 who had never tested positive, HTC uptake was 88.5% in villages and 79.5% in urban areas (p < 0.001). Overall coverage after first visit was 61.8% in rural villages and 61.5% in urban areas (*p* = 0.74).

### Coverage after second-visit

**Fig. 1:**
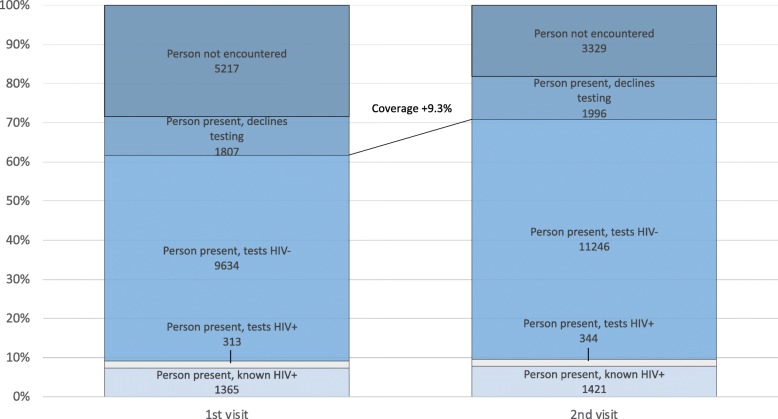
Testing coverage after first and second visit

Of the 6655 households surveyed during first visit, 2004 (30.1%) had absent members and were re-visited on the following weekend. Of the 5217 household members absent during the first visit, 1890 (36.2%) were encountered during the second visit. Just over half of these were children who had mostly been at school (Table 1). Of the 1890 newly present members at the second visit, 56 were known to be HIV positive. Among those who had never tested HIV positive, 1647 (90.4%) took up HTC (urban 84.0%, rural 93.7%, *p* < 0.001). With the second visit, overall HTC coverage increased to 71.0% (Fig. 1), 69.4%% in urban and 71.9% in rural areas (*p* < 0.001).

**Table 1:** Age and gender of household members

	N	Encountered at 1st visit	Encountered at 2nd visit	Not encountered
n	18,336	13,119	1890	3327
Median Age (IQR)	25 (12–45)	28 (14–52)	14 (9–25)	19 (11–34)
Women ≥15 years	7875	6671 (50.9)	459 (24.6)	745 (22.5)
Men≥15 years	4807	3029 (23.1)	436 (23.3)	1342 (40.5)
Children < 15 years	5604	3398 (25.9)	978 (52.2)	1228 (37.0)
Age/sex missing	50 (0.3)	21 (0.2)	17 (0.9)	12 (0.4)

Table 1 shows the age and gender distribution of all household members according to their presence during the visits. Table 2 shows the reasons for absence in household members not encountered.

**Table 2:** Reasons for not being at home at first and second visit among household members not encountered (29 are not included in the table due to missing information about sex or age)

Location of absent household members	First visit	Second visit
Women ≥15 years	1204	745
– At school	318 (26.4)	197 (26.4)
– Work, commuting daily	148 (12.3)	115 (15.4)
– Work, home on weekends	44 (3.7)	36 (4.8)
– Work in South Africa	84 (7.0)	80 (10.7)
– In the fields/herding	36 (3.0)	22 (3.0)
– Single day absence (not work-related)	386 (32.1)	256 (34.4)
– On holidays	23 (1.9)	20 (2.7)
– No information	165 (3.7)	19 (2.6)
Men ≥15 years	1778	1342
– At school	277 (15.6)	193 (14.4)
– Work, commuting daily	178 (10.0)	140 (10.4)
– Work, home on weekends	69 (3.9)	59 (4.4)
– Work in South Africa	250 (14.1)	231 (17.2)
– In the fields/herding	465 (26.2)	373 (27.8)
– Single day absence (not work-related)	417 (23.5)	322 (24.0)
– On holidays	19 (1.1)	17 (1.3)
– No information	103 (5.8)	7 (0.5)
Children	2206	1228
– At school	1657 (75.1)	981 (79.9)
– Work, commuting daily	5 (0.2)	2 (0.2)
– Work, home on weekends	2 (0.1)	2 (0.2)
– Work in South Africa	3 (0.1)	3 (0.2)
– In the fields/herding	85 (3.9)	62 (5.1)
– Single day absence (not work-related)	244 (11.1)	161 (13.1)
– Away on holiday	14 (0.6)	12 (1.0)
– No information	196 (8.9)	5 (0.4)

### HTC uptake and HIV prevalence

If encountered at home, overall uptake was 85.3%. Table 3 displays likelihood of being encountered at home and HTC uptake in children, adult women and men. Men and children were less likely to be encountered at home. But if encountered, uptake of HTC was similar.

**Table 3:** Likelihood of being encountered at home and HTC uptake according to age and gender

	Total n	n (%)	Odds-ratio (95%CI)	*p*-value
Total HH members	18,286^a^			
Ever encountered at home				
– Women ≥15 years	7875	7130 (90.5)	1	
– Men ≥15 years	4807	3465 (72.1)	0.27 (0.24–0.30)	< 0.001
– Children < 15 years	5604	4376 (78.1)	0.37 (0.33–0.41)	< 0.001
HTC Uptake if encountered and not known HIV positive	13,554	11,592 (85.3)		
– Women ≥15 years	6119	5144 (84.2)	1	
– Men ≥15 years	3130	2613 (83.5)	0.95 (0.84–1.07)	0.38
– Children < 15 years	4314	3816 (88.5)	1.44 (1.28–1.62)	< 0.001
HTC coverage after 2 visits	18,286^a^	12,991 (71.0)		
– Women ≥15 years	7875	6165 (78.3)	1	
– Men ≥15 years	4807	2948 (61.3)	0.44 (0.41–0.48)	< 0.001
– Children < 15 years	5604	3878 (69.2)	0.62 (0.58–0.67)	< 0.001

^a^50 individuals were excluded from the table due to missing information on gender and/or age

HIV prevalence among those tested was 0.6, 4.2 and 4.0% in children, adult women and adult men, respectively (Table 4). Considering persons encountered at home who were already known to be HIV positive and individuals newly diagnosed during home-based HTC, the prevalence among adults aged 15–49 years was 18.8, 21.6% in women and 13.0% in men.

**Table 4:** HTC uptake and HIV prevalence among household members encountered at home who were not known to be HIV positive

	N^a^	HTC uptake %	OR (95%CI)	p-value	Tested HIV^+^ (%)	OR (95%CI)	*p*-value
Women ≥15 years	6110	84.2			4.2		
Never tested	1499	81.3	Ref		4.3		
< 12 months ago	2903	82.2	1.06 (0.90–1.25)	0.46	3.1	0.72 (0.50–1.03)	0.07
≥ 12 months ago	1677	91.2	2.37 (1.92–2.94)	< 0.001	6.0	1.42 (1.00–2.01)	0.05
Men ≥15 years	3130	83.5			4.0		
Never tested	1188	82.0	Ref		4.6		
< 12 months ago	901	77.8	0.77 (0.62–0.96)	0.02	2.7	0.58 (0.33–0.99)	0.05
≥ 12 months ago	1016	91.2	2.29 (1.76–2.98)	< 0.001	4.4	0.96 (0.62–1.48)	0.84
Children < 15 years	4314	88.5			0.6		
Never tested	2650	89.3	Ref		0.8		
< 12 months ago	863	85.1	0.68 (0.55–0.85)	0.001	0	–	
≥ 12 months ago	769	91.0	1.20 (0.91–1.56)	0.20	0.3	0.35 (0.08–1.53)	0.16

^a^From the total number, time since last HIV test was missing for 31 women, 25 men, and 32 children

### First-time testers

Among all individuals encountered, 5337 (39.4%) were first time testers, and 3462 (25.5%) had tested ≥12 months ago. There was a significant difference in the share of first-time testers between persons tested at first and second visit (*p* < 0.001): at first visit 34.8% had never tested before (second visit: 46.9%), 31.6% had their last HIV test ≥12 months ago (second visit: 28.1%) and 33.7% had tested < 12 months ago (24.9%). First time testers were more likely to uptake HTC compared to those recently tested (OR: 1.30; 95%CI: 1.16–1.44) and less likely than those tested > 12 months ago (OR: 0.57; 95%CI: 0.50–0.66) (Table 4).

### Awareness of HIV status and ART-coverage

Out of the 1765 individuals encountered who were HIV positive, 1421 (80.5%) already knew their status before the home-based HTC campaign and 1241 (70.3%) were already on ART.

### Cost estimates

Overall direct costs of the HTC campaigns were USD 137,510; USD 38,322 for senior staff (nurses and campaign organizers); 16,738 for lay-counsellors and drivers; 26,050 for food and accommodation during the campaign as well as small incentives, such as bags, coats and hats; 30,500 for transport (fuel, vehicle repair and maintenance); 25,900 for HIV test kits, IT cost, purchase of tablets and small equipment, such as gloves, needles, etc.

Overall cost per person tested for HIV, and per person newly tested HIV positive was USD 11.8 and 399.7, respectively. Costs were higher during weekend catch-up visits. Per person testing cost were 10.5 and 20.0 during week and weekend days respectively. Similarly, costs per person tested HIV positive was lower during weekdays (335 versus 1056). Differences in cost between weekdays and weekends were driven by a lower average number of persons tested per lay-counsellor on weekend days (3.0 vs 6.6) resulting in higher staff-cost relative numbers tested.

## Discussion

This study tested the effectiveness of a weekend catch-up visit to increase home-based HIV testing coverage in rural and urban areas in Lesotho, Southern Africa. After a first home-based HTC visit during a work-day, overall coverage was 62% and increased to 71% after a catch-up visit during the following weekend. This increase came at considerable additional cost: Testing a person cost about twice as much during catch-up visits, and cost per person newly tested positive was three times higher due to fewer persons tested per health care worker on weekends. During the catch-up visit, 47% were first-time testers, compared to 35% at the visit. While HTC uptake was similar between women and men, coverage was significantly lower in men due to a higher share of male household members who were absent at both visits. In urban areas household members were more likely to be encountered at home but uptake was lower, resulting in a slightly lower HTC coverage in the urban areas. Although, in this study, targeted catch-up visits improved testing coverage, it still fell short of the targeted 90% testing coverage, mainly because many household members were absent at both visits.

To our knowledge, this is the first study quantifying the added value of targeted catch-up visits after a first round of home-based HTC. For assessing coverage, we did not rely on census data or extrapolated estimates but directly censored household-members during the campaign. We defined as household members any person sleeping at least two nights per month in that household. This broad definition may have led to a lower HTC coverage compared to other studies. In the SEARCH project HTC coverage was studied among “stable residents”, defined as those, “living in the community for at least 6 months in the past year”. In Ugandan and Kenyan areas together, the SEARCH project achieved among adults a 89% HTC coverage, 86% among men and 92% among women [18]. Testing coverage among adolescents was 88% [19]. Another project, conducted in Uganda, calculated coverage based on official census data. In that study, during a period of 6 months, 66 trained community health workers provided home-based HTC to over 40′000 individuals reflecting an estimated HTC coverage of 69.4% at the end of the project [20].

Whereas in our study direct cost per person tested for HIV was USD 11.8 USD and USD 399.7 per person tested and per person tested HIV positive, respectively. Asiimwe et al. reported from Uganda costs of USD 3 and 136 per person tested and person tested positive, respectively [20]. In the SEARCH, project home-based HTC was more expensive than community health campaigns and came at a cost of USD 31.7 per adult tested and 298.5 per adult tested HIV positive [21]. In line with these reports, our study shows that home-based HTC is a cost-intensive approach. On the other hand, home-based HTC remains a good strategy to reach individuals who might not seek testing at a health facility. In our study, close to 40% indicated they were testing for the first time, and 25% stated that their last HIV test was more than 12 months ago. As demonstrated in immunization programs previously, increasing coverage from an already high coverage to the targeted 90% immunization coverage comes at a higher cost per percentage than increasing from a lower level to 80% [22]. Similarly, to close the gap from 80 to 90% HIV testing coverage will need more resources. This is particularly true for remote rural areas, where finding people living with HIV who are not on treatment is time- and resource intensive.

For those encountered at home, uptake of HTC was 85.3%. This is lower than in a previous study conducted in the same setting, where 92.5% accepted HTC [12]. Possible explanations are that the earlier study provided multi-disease testing and that it was conducted only in rural areas. In the current study HTC uptake was substantially lower in urban areas, resulting in a lower overall coverage. Several studies, using a multi-disease approach, report uptake rates for HIV testing over 90% [23–25]. A door to door HIV testing campaign conducted in neighboring KwaZulu Natal reports uptake rates of 76 and 86% in men and women respectively [26]. Of note, in our study, HTC uptake was similar in men and women (Table 3) – in contrast to many studies in similar settings, where generally men showed lower acceptance rates [18, 26]. We are not able to comprehensively explain the similar HTC acceptance rates among women and men, but may assume that this is a characteristic of our setting, as we found the same result in a the previous study [12]. Men were, however, encountered less often at home, leading to a significantly lower HTC coverage among men. They were mostly absent due to travel, work in the field or work in neighboring South Africa (Table 2). During the SEARCH project in Uganda and Kenya, the fact that men’s labor opportunities often require extended absences from home was frequently cited as a reason for lower testing coverage in men [27]. However, absence on the day of HTC may also have been caused by men not wanting to test for HIV. As communities were informed about the HTC campaign in advance, it is possible that some household members chose not to be home on the day of the campaign. In order to target men who were absent during home-based HTC, social network interventions, self-testing and testing at the workplace are promising interventions [28]. Moreover, the recent initiative of male-friendly clinics where male health care workers provide services has shown some first promising results [29]. Another approach may be to leave for household members who are absent an oral self-test kit [30]. As for men, it was also difficult to reach school-aged children. One approach might be to provide large-scale school-based HTC. In a survey in South Africa, over 90% of parents supported HIV testing at schools [31].

Despite Lesotho having the second-highest HIV prevalence in the world, yield of new HIV diagnoses in our study was low with 4.2 and 4.0% in women and men respectively. However, considering all those encountered who already knew about their positive HIV status, adult prevalence in the population assessed was 18.8%, which is similar to the reported adult HIV prevalence in Butha-Buthe of 17.8% [15]. In line with the Lesotho population-based impact assessment (LePHIA) [15], our findings indicate that already in 2016 about 80% of HIV infected persons knew their status and 70% are taking ART. The encouraging fact that Lesotho is progressing towards the 90–90-90 targets, decreases the yield of newly tested positive individuals during home-based testing. Considering the yield of new HIV diagnoses, home-based testing may thus not be an efficient approach anymore [32]. However, several studies indicated that the benefit of home-based HTC should not only be seen in numbers newly found HIV positive as a negative HIV test may increase awareness and reduce risk-behavior [33]. Moreover, as indicated by high shares of first-time testers in our study, home-based HTC remains a valid approach to test persons who might not access testing otherwise. Furthermore, home-based HTC may serve to re-engage individuals who are known HIV positive but who never linked to care or dis-engaged from care. Furthermore, home-based HIV testing may be combined with services addressing other conditions, such as mother-and-child health, tuberculosis, mother-and-child health, and screening for viral hepatitis and other sexually transmitted diseases.

This study has several limitations. First, for calculating coverage, we used a broader definition of household member than earlier studies, and censoring of absent household members relied on reporting from those members who were found at home. Second, household members not encountered may well have been tested for HIV on another occasion, i.e. at their workplace in South Africa or at one of numerous testing-points within Lesotho. Finally, previous testing history relied on interviews and on information found in the health booklets of the household members.

## Conclusions

In summary, during home-based HIV testing campaigns, a second catch-up visit during the weekend increased testing coverage in the whole community by about 9% and reached more first-time testers. However, the weekend catch-up visit increased cost per person tested and per person newly tested HIV positive considerably. Overall, even with a catch-up visit, testing coverage in the assessed communities fell short of the targeted 90%. More research is needed about how to design more cost-effective HTC campaigns *with a particular emphasis on providing different approaches for different population groups*, e.g. by combining community- and home-based testing or introducing self-testing for those household members not encountered at home or refusing to test.

## Supplementary information

**Additional file 1: Table S1.** Unit cost used for cost-calculations. Salaries include additional cost as per legal requirement in Lesotho. Transport cost included fuel, vehicle insurance, tire replacement, car servicing and repair but not the purchase of the vehicle.

## Data Availability

Upon request to the co-author responsible for data-management and statistical analysis (TRG).

## Abbreviations

AIDS: Acquired Immunodeficiency Syndrome
CI: Confidence Intervall
HB-HTC: Home-Based HIV Testing and Counselling
HH: Household
HIV: Human Immunodeficiency Virus
HTC: HIV Testing and Counselling
IQR: Interquartile Range
OR: Odds Ratio
UNAIDS: Joint United Nations Programme on HIV/AIDS
WHO: World Health Organization
