# Supplementary material for: Effect and cost of two successive home visits to increase HIV testing coverage: a prospective study in Lesotho, Southern Africa
Source: BMC Public Health. 2019 Nov 1;19:1441. doi: 10.1186/s12889-019-7784-z (PMC6825349; doi:10.1186/s12889-019-7784-z)
Supplement: Supplementary file 1 — Additional file 1: Table S1. Unit cost used for cost-calculations. Salaries include additional cost as per legal requirement in Lesotho. Transport cost included fuel, vehicle insurance, tire replacement, car servicing and repair but not the purchase of the vehicle. [file 12889_2019_7784_MOESM1_ESM.docx]

| Item | cost per unit | n | total |
| --- | --- | --- | --- |
| Human Resources Cost |  |  |  |
| - Salary professional counsellor (7 months) | 5972 | 3 | 17916 |
| - Salary lay-counsellors (7 months) | 842 | 15 | 12630 |
| - Salary nurses (7 months) | 6801 | 3 | 20403 |
| - Salary 2 drivers (7 months) | 2049 | 2 | 4098 |
| - Overall cost for lunch/perdiems/allowances, etc. | 24’250 |  | 24’250 |
| - Equipment for counsellors (bags, coats, hats, etc....) | 1800 |  | 1’800 |
| Transport Cost | 30500 |  | 30’500 |
| Equipment |  |  |  |
| - Purchase and set-up of tablet computers and installation | 1500 |  | 1’500 |
| - Programming of specific software and database | 10000 |  | 10’000 |
| - HIV test kits | 1 | 11000 | 11000 |
| - Other equipment (gloves, finger-prick, cotton, etc.) | 1000 |  |  |
| Other Cost |  |  |  |
| - Meetings and trainings | 2400 |  | 2400 |
